# Improving investment in chronic disease care in Sub-Saharan Africa is crucial for the achievement of SDG 3.4: application of the chronic care model

**DOI:** 10.1186/s13690-023-01181-5

**Published:** 2023-09-14

**Authors:** Hubert Amu, Theodora Yayra Brinsley, Frank Oppong Kwafo, Selasi Amu, Luchuo Engelbert Bain

**Affiliations:** 1 Department of Population and Behavioural Sciences, School of Public Health, University of Health and Allied Sciences, Hohoe, Ghana; 2 Department of Epidemiology and Biostatistics, School of Public Health, University of Health and Allied Sciences, Hohoe, Ghana; 3 Department of Midwifery, School of Nursing and Midwifery, University of Health and Allied Sciences, Ho, Ghana; 4 Department of Psychology, Faculty of Humanities, University of Johannesburg, Johannesburg, Auckland Park, South Africa; 5 International Development Research Centre, IDRC, Ottawa, Canada

**Keywords:** Chronic non-communicable diseases, Sustainable development goals, Chronic care model, Sub-saharan Africa

## Abstract

Over 41 million people die of chronic non-communicable diseases (CNCDs) each year, accounting for 71% of all global deaths. The burden of CNCD is specifically a problem in sub-Saharan Africa (SSA) since CNCDs are largely a leading major cause of mortality in the sub-region. While the disease burden and mortality from chronic non-communicable diseases (CNCDs) have reached an epidemic threshold in sub-Saharan Africa (SSA), health systems, policy-makers and individuals still consider CNCDs to be uncommon and, therefore, do not give its management the required attention. In sub-Saharan Africa (SSA), effectively addressing the growing burden of CNCDs will require comprehensive measures that incorporate both curative and preventive interventions, towards achieving the Sustainable Development Goal (SDG) 3.4 target of reducing by one-third premature mortality from CNCDs through prevention and treatment and the promotion of mental health and well-being by the year 2030. In this commentary, we adopt the Chronic Care Model (CCM) to discuss how improved investment in Chronic Disease Care is crucial in achieving the SDG target in SSA. At the health systems level of the CCM, we propose that countries in SSA should increase the proportion of their annual budgets allocated to health in line with the Abuja Declaration of 2001. Social health insurance should also be adopted by all countries and effectively implemented. At the community level, we propose intensified community-based health education, the formation of peer support groups and the implementation of community-based policies that promote healthy eating and physical activity.

## Background

Globally, CNCDs have become a major burden to health systems. Over 41 million people die of these diseases yearly, accounting for 71% of all global deaths [1, 2]. They are currently the primary cause of mortality, and their significance, compared to injuries and communicable diseases, is estimated to increase in the next two decades globally [3]. These diseases include cardiovascular disease (CVD), cancer, diabetes mellitus, sickle cell disease (SCD), glaucoma, hypertension, cerebrovascular accident (stroke), chronic heart disease, chronic lung disease, and, asthma. They tend to be of long duration and result from a combination of environmental, physiological, behavioral, and genetic factors [4].

Countries in SSA are currently undergoing a rapid epidemiological transition marked by a surge in the disease burden of CNCDs, and have expectedly overtaken infectious diseases as a major contributor to morbidity and mortality [4, 5]. SSA is expected to see one of the largest increases in mortality due to CNCDs globally and an estimated increase in deaths by 46% of all deaths by the year 2030 if immediate measures are not taken [5]. The rising burden of CNCDs in SSA between 50% and 88% of deaths includes factors such as lifestyle changes, rapid urbanization, population growth, rising life expectancy, and epidemiological transition [6].

The 2030 Agenda for Sustainable Development recognizes CNCDs as a major challenge to sustainable development. CNCDs prevail in the sub-region against the backdrop that meeting the needs of the ever-increasing population in SSA is critical to achieving SDG 3, which seeks to ensure healthy lives and promote well-being for all at all ages. Specifically, target 3.4 seeks to reduce, by one-third, premature mortality from CNCDs through prevention and treatment and the promotion of mental health and well-being. by the year 2030 [7]. Countries in SSA are, however, still far from achieving any milestones regarding achievement of the SDG 3.4. For example, at the country level, our literature search did not reveal any universal levels of patients being screened for NCDs like diabetes and hypertension, and successfully put on treatment as required by the year 2030.

This commentary is underpinned by the CCM, which was developed by the MacColl Institute for Healthcare Innovation at Group Health Cooperative in 1992 [8]. The CCM is a well-established and validated framework that explains a comprehensive approach to caring for the chronically ill, that supports increased functional and clinical outcomes. In the chronic care paradigm, care is offered in a primary healthcare setting with the goal of bringing together the patient, provider, and system interventions required to achieve the overarching goal of improving chronic illness care. The key constructs of the model are the health system and the community, which also have sub-constructs such as self-management support, decision support, and clinical information systems [8]. The CCM has six constructs which are; the community, the health system, self-management support, delivery system design, decision support, and clinical information. But these six constructs can be categorized under two key constructs, the health system (delivery system design, clinical information system, and the health system) and the community (self-management support, the community, and decision support). These elements are designed to work in collaboration to strengthen the provider-patient relationship and improve the health outcomes of people living with CNCDs. The CCM has, thus, been adopted to explain how improving investment in chronic disease care is crucial for the achievement of SDG 3.4. There is currently a paucity of literature on the nexus between effectively managing CNCDs and how accelerates progress toward the attainment of the SDGs by the year 2030. This commentary could, therefore, be instrumental in informing policy and practice regarding CNCD care in SSA.

## Health systems

The delivery system design and clinical information are categorized under the key construct ‘health systems’. The health system forms the backbone of the management of CNCDs in SSA. Wagner (2001) [8] argued that to ensure effective management which improves the health outcomes of people living with CNCDs, there is a need for a strong health system that provides safe and high-quality care. The health system should have a dedicated and motivated team of health professionals who are well-positioned to support improvement strategies and encourage open and systematic handling of errors through the provision of quality care. The management of CNCDs under the health systems tenet of the CCM includes general services provided by health professionals irrespective of the CNCDs presented by patients. This includes checking vital signs (such as blood pressure, blood sugar, oxygen levels, temperature, pulse and respiration, laboratory tests, history taking, general education, and counseling on the conditions) to determine the health status of clients.

There are also specific services provided to patients based on the CNCDs presented and the stage of the conditions upon presentation. These include medical and surgical procedures including chemotherapy, physiotherapy, dialysis, surgeries, and prescription of medications specific to the CNCDs. The increasing prevalence of CNCDs with its rapidly increasing demand on health systems makes it nearly impossible for the health systems of SSA countries to keep up with the provision and care for the management of CNCDs management and services as expected [8]. This comes against the backdrop that even before the upsurge of CNCDs, the health systems in SSA were constrained by the non-availability or inadequacy of health-related human resources, logistics (equipment, medicines, and laboratory supplies), and funding, which hindered the provision of optimum patient care. The countries, therefore, depended largely on international donor support.

To address the challenges with the health systems, we propose various multi-sectoral investments needed to accelerate progress towards the achievement of SDG 3.4. In April 2001, all 53 heads of state of the African Union met in Abuja, Nigeria, and ratified a declaration to allocate at least 15% of their annual budgets to their respective health sectors [9]. Since the Abuja Declaration, the various countries have not lived up to the treaty signed. In 2021 for instance, countries like Ghana, Nigeria, South Africa, Kenya, and Tanzania allocated 9.1% (up from 7.7% to 2020), 4.6% (up from 4.4% to 2020), 9.3% (up from 8.5% to 2020), 5.5% (down from 6.0% to 2020), and 7.1% (up from 5.8% to 2020) respectively of their annual budgets to the health sector. In SSA countries, domestic health financing sources come from the general government (not necessarily limited only to ministries of health) and many exclude grants and other forms of official development aid (ODA). Increasing the priority given to health in the annual budget is crucial for the achievement of SDG 3.4 [9]. Thus, an increase in the proportion of the total government budget allocated to health in line with the Abuja Declaration would help the health systems of SSA countries to adequately provide care to patients with CNCDs. This is because access to healthcare in many SSA countries is still a challenge, especially in last-mile communities. With increased funding for the health sectors, infrastructure, logistics, and human resources needed to ensure optimal healthcare delivery could be realized towards achieving SDG target 3.4. In addition, an improvement in geographical and financial access by the various health authorities in SSA will go a long way to making healthcare delivery very effective and efficient as people will not travel long distances to access healthcare, and out-of-pocket and copayment will also stop, thereby reducing high morbidity and mortalities with its associated complications among populations in SSA.

While some SSA countries like Ghana, South Africa, and Kenya have implemented national policies for the control and prevention of CNCDs, most of the countries in the sub-region including Sierra Leone, Chad, and Somalia have not yet developed such policies. We recommend that the countries which currently do not have such policies should implement them to ensure holistic prevention and management CNCDs. The policies should generally seek to reduce the incidence, prevalence, and exposure of people to CNCD risk, reduce morbidity associated with the disease, and improve the overall quality of life of persons living with CNCDs. They should also focus on investments such as primary prevention and clinical care including early detection, provision of treatment services, health system strengthening involving the training of health professionals, and the development of human resource capacity.

Social health insurance has been shown as a crucial investment in improving the treatment of CNCDs [10]. In SSA, while countries like Ghana, Kenya, Nigeria, and Tanzania have successfully implemented National Health Insurance (NHI) policies to improve the health status of their residents [11], countries like Central Africa Republic, Chad, Somalia, and South Sudan have not yet implemented them. Access to basic healthcare in Chad, for instance, is limited while the country has a high prevalence of CNDs and other diseases [12]. Azetsop and Ochieng [12], have, however, shown that access to national health insurance schemes can help to mitigate such disease risks. To accelerate progress towards the achievement of SDG 3.4 through the elimination of out-of-pocket payments, we recommend that the countries yet to implement national health insurance schemes should do so to ease the financial burden of accessing and utilizing healthcare. For countries currently implementing health insurance but have separate schemes for various groups, we recommend that the schemes should be harmonized to help maximize the size of their risk pools and increase the confidence of potential subscribers in the system [2].

## Community

The sub-constructs; self-management support, and decision support can be categorized under the key construct ‘the community’.

Community as a tenet of the CCM entails Advocacy Communication and Social Mobilization for policies that improve CNCD care [13]. Self-management support involves support provided by health professionals and caregivers (family, friends, and neighbors) through goal setting. It also includes action planning, coping, follow-up, and preparing CNCD patients to socially and financially manage their conditions when they are in their respective communities (at home) [13]. In Ghana, self-management of CNCDs by patients involves management processes such as self-restriction (including diet restrictions), exercise, personal first aid, and the use of anthropometric equipment to monitor health status which patients adopt in managing their conditions at home [14]. The CCM recognizes caregiver roles such as emotional support, staying with the patient, helping patients with exercises, and reminding them of their self-restrictions as central in the self-management of CNCDs by patients.

To confront chronic disease in the community in SSA, we propose several investments that when implemented can be crucial towards the achievement of SDG 3.4. Education and raising awareness about chronic disease and its risk factors are key to the prevention and management of CNCDs. Community-based health education programs can be used to teach people about healthy lifestyle choices, such as proper nutrition, regular physical activity, and avoiding tobacco use [4]. Evidence of this is seen in Ghana, where community-based healthcare providers, educate community members on health promotion activities like good dietary practices, the importance of physical activity, and reduction in tobacco and alcohol consumption. This is done at the Community-based Health Planning and Services (CHPS) which should not only bother on health education but also to provide holistic basic health care to all manner of persons living in such environs. e.g. immunization, water and sanitation, Antenatal Care, postnatal Care, Family Planning, supervising delivery and treatment for minor ailments among others [15]. Ensuring access to healthcare services is crucial for individuals with chronic conditions. This includes providing preventive services such as screenings, check-ups, and vaccinations, as well as disease management services such as medication management and follow-up care [16]. Support groups can help individuals with chronic conditions and their families connect with others who are going through similar experiences. This can provide emotional support and practical advice for managing the condition [5]. Improved investments in public health policies can have a significant impact on chronic disease prevention and management. For instance, implementing policies that promote healthy eating and physical activity, as well as policies that regulate the marketing and sale of tobacco products, can help prevent chronic diseases. Research and innovation into the causes, prevention, and treatment of chronic diseases can lead to new discoveries and innovations that improve outcomes for individuals with these conditions [11].

Overall, confronting chronic disease in the community requires a comprehensive approach that involves education, access to healthcare, support groups, policy changes, and research and innovation. By working together, communities can reduce the burden of chronic disease and improve the health and well-being of their residents towards the achievement of SDG 3.4.

## Conclusion

In this paper, we posit that improving investment in chronic disease care in SSA is crucial for the achievement of SDG 3.4. The Chronic Care Model provides a comprehensive framework for addressing the complex needs of patients with chronic diseases. To accelerate progress towards meeting SDG 3.4 in SSA, grounded on the CCM, there is a need for increased investment in healthcare financing, adoption and effective implementation of social health insurance, and increased community-based health education. There should also be sustained training of health professionals and effective implementation of public health policies that prioritize chronic disease care. Through these investments, SSA countries can reduce the prevalence of CNCDs, improve the quality of care provided to patients with the diseases, reduce catastrophic out-of-pocket healthcare costs, and ultimately achieve SDG 3.4 which aims to reduce premature mortality from CNCDs by the year 2030.

## Data Availability

All data used in the commentary are included in the main manuscript file.
